# Late-onset hypophysitis after discontinuation of nivolumab treatment for advanced skin melanoma: a case report

**DOI:** 10.1186/s12902-021-00854-y

**Published:** 2021-09-20

**Authors:** Sofia Antoniou, Georgios Bazazo, Ludwig Röckl, Marios Papadakis, Christian Berg

**Affiliations:** 1 Department of Internal Medicine, Angiology, Endocrinology and Diabetology, Protestant Hospital Mettmann, Gartenstrasse 4-8, 40822 Mettmann, Germany; 2 Department of Surgery II, University of Witten-Herdecke, Heusnerstrasse 40, 42283 Wuppertal, Germany; 3 Department of Infectious Diseases, Department of Nephrology, University Hospital Essen, University Duisburg Essen, Duisburg, Germany

**Keywords:** Hypophysitis, Nivolumab, Skin melanoma, Cancer immunotherapy

## Abstract

**Background:**

Nivolumab is an anti-programmed cell death protein 1 antibody, typically used as cancer immunotherapy agent. Despite multiple clinical benefits it might cause autoimmune-related side-effects, often involving the endocrine system. To our knowledge, this is the first case of nivolumab-induced hypophysitis manifesting several months after treatment discontinuation.

**Case presentation:**

We, herein, report a 53-year-old patient with hypophysitis and isolated adrenocorticotropic hormone deficiency, who presented with recurring syncopal episodes and persistent mild hyponatremia. The performed challenged tests were consistent with secondary adrenal insufficiency, while responses of other anterior pituitary hormones were preserved. Magnetic resonance imaging revealed thickened pituitary stalk, consistent with hypophysitis. The patient’s condition gradually improved after administration of hydrocortisone, with normalization of sodium and glucose-levels. The related literature is discussed.

**Conclusions:**

We conclude that even after discontinuation of nivolumab, isolated adrenal insufficiency can occur. Therefore, in case of administration of such agents, clinical assessment, and routine monitoring of blood pressure, sodium-, glucose-levels, pituitary hormones as well as magnetic resonance imaging are needed to identify such conditions and prevent an adrenal crisis.

## Introduction

Hypophysitis is a chronic or acute inflammation of the pituitary gland. It can resolve spontaneously and is often refractory to treatment [1]. It is a rather rare disease with an exponentially increasing annual incidence, mostly due to increasing awareness and knowledge [2]. Several classifications, based on its etiology, have been suggested. While primary hypophysitis is a result of pituitary inflammation, secondary hypophysitis refers to cases of systemic infections, pituitary adenomas and medication [3–5].

Increasing evidence suggests hypophysitis may be associated with immunotherapy (interleukin 2, interferon) and medications targeting cytotoxic T-lymphocyte antigen-4 (CTLA-4) or programmed cell death 1 (PD-1) [6]. The latter, also known as anti-PD-1 monoclonal antibodies (mAbs), have become the standard frontline treatment for advanced BRAF-wild-type melanoma.

Nivolumab has recently been approved in Western countries for the treatment of advanced melanoma [7]. Few published cases, mostly referring to ipilimumab, which has been longer used, suggest an association with endocrine immune-related adverse events, such as hypophysitis and thyroid dysfunction [8]. Only few cases report nivolumab induced-hypophysitis, mostly in patients still undergoing treatment [9].

We herein report a case of nivolumab-induced hypophysitis in a 53-year-old male patient. To our knowledge, this is the first case of nivolumab-induced hypophysitis manifesting several months after treatment discontinuation.

## Case presentation

A 53-year-old male patient was admitted to our Department due to syncopal episodes over the past few months. He was also suffering from fatigue and mild anorexia. Prior to referral he had consulted a cardiologist, who performed several diagnostic tests that failed to reveal a certain cause. Blood pressure was normal (systolic blood pressure, SBP: 103 mmHg, diastolic blood pressure, DBP: 73 mmHg). Unfortunately, a 24-h-ambulatory blood pressure test was not performed, but orthostatic hypotension was found (SBP: 95 mmHg, DBP: 66 mmHg).

At that time, he was not receiving any regular medication. Fourteen months earlier he was diagnosed with malignant melanoma with right axillary lymph node involvement. After radical lymph node dissection, he received treatment with nivolumab (about 21 cycles). Histology was consistent with a BRAF-Wild-Type Mutation. The last cycle of nivolumab treatment was given about 6 months before admission. Cancer tumor staging showed no metastasis or local invasion and the last magnetic resonance imaging (MRI) was normal. A previous endocrine disorder was not known. The patient was not smoking or consuming alcohol on a regular basis. His level of consciousness was normal. Severe headache, diarrhoea or abdominal pains were not reported. Physical examination showed no skin pigmentation or vitiligo. The family history was negative for any endocrine disorders or cardiovascular disease, as far as the patient was concerned.

Laboratory tests revealed serum hyponatremia (125 mmol/l), which was rather persistent, according to the patient’s records. Almost 3 weeks prior to amission to our department, sodium levels of 123 and 125 mmol/l were documented from the cardiologists, who performed the initial examinations. The patient’s symptoms aggravated over the past month, therefore it is unlikely that hypophysitis has previously been overseen. Moreover, sodium levels after completing the treatment with nivolumab were found normal in the routine examination by the general practitioner. Oncologists did not document any hyponatriemia either. We found an asymptomatic hypoglycemia (58 mg/dl) at the time of admission. Blood glucose (BG) was further regularly controlled and found normal. Serum-free thyroxine levels (FT4), serum thyroid-stimulating hormone (TSH), as well as Anti-thyroid peroxidase (TPOAb) and anti-thyroglobulin (TgAb) antibodies were at normal range, suggesting a normal thyroid function. No goiter was observed. There was no indication of gonadal insufficiency or hyperprolactinemia. Serum und Urine Osmolality were normal; in the absence of polydipsia and polyuria a diabetes insipidus was excluded (Table 1). Inflammatory markers such as CRP protein were normal and tuberculosis as well as sarcoidosis were excluded by the oncologists.

**Table 1:** Laboratory measurements in our patient

Parameter	Value	Normal range
Hb (g/dl)	12.3	14-16.5
BG (mg/dl)	88	74–106
Sodium (mmol/l)	125	136–145
Potassium (mmol/l)	4.7	3.5–5.1
GFR (ml/min)	107.5	80
TPOAb (U/ml)	37.5	< 59
TgAb (U/ml)	25	
TSH (uIU/ml)	2.01	0.3-4.0 uIU/ml
FT4 (ng/dl)	1.6	0.9–1.8
Basal ACTH (pg/ml)	1.0	< 63
Serum Osm. (mOsm/l)	253	280–300
Urin Osm. (mOsm/l)	259	50-1400
Aldosterone (ng/l)	77	11.7–236
Renin (ng/l)	5.7	2.8–39.9
Testosterone (ng/ml)	272	129–767
FSH (IU/l)	3.3	1.5–12.4
LH (IU/l)	7.3	1.7–8.6
SHBG (mmol/l)	133.4	-
Prolactin (ng/ml)	5.8	2.5–17
DHEAS (ug/dl)	29.1	35–436
IGF-1 (ng/ml)	242.8	125–290

*Hb* Haemoglobin, *BG* blood glucose, *GFR* Glomerular filtration rate, *TPOAb* anti-thyroid peroxidase antibodies, *TgAb* antithyroglobulin antibodies, *TSH* thyroid stimulating hormone, *ACTH* Adrenocorticotropic hormone, *FSH* follicle-stimulating hormone, *LH* Luteinizing hormone, *SHBG* Sex hormone binding globulin, *DHEAS* Dehydroepiandrosterone sulfate, *STH* Somatotropin hormone, *IGF-1* insulin-like growth factor 1, *FT4* free thyroxine, *Osm* Osmolarity

To further clarify the cause of the mild anaemia -without eosinophilia- we performed a routine control of the gastrointestinal tract. Endoscopy findings were consistent with chronic, Helicobacter Pylori-negative gastritis.

**Fig. 1:**
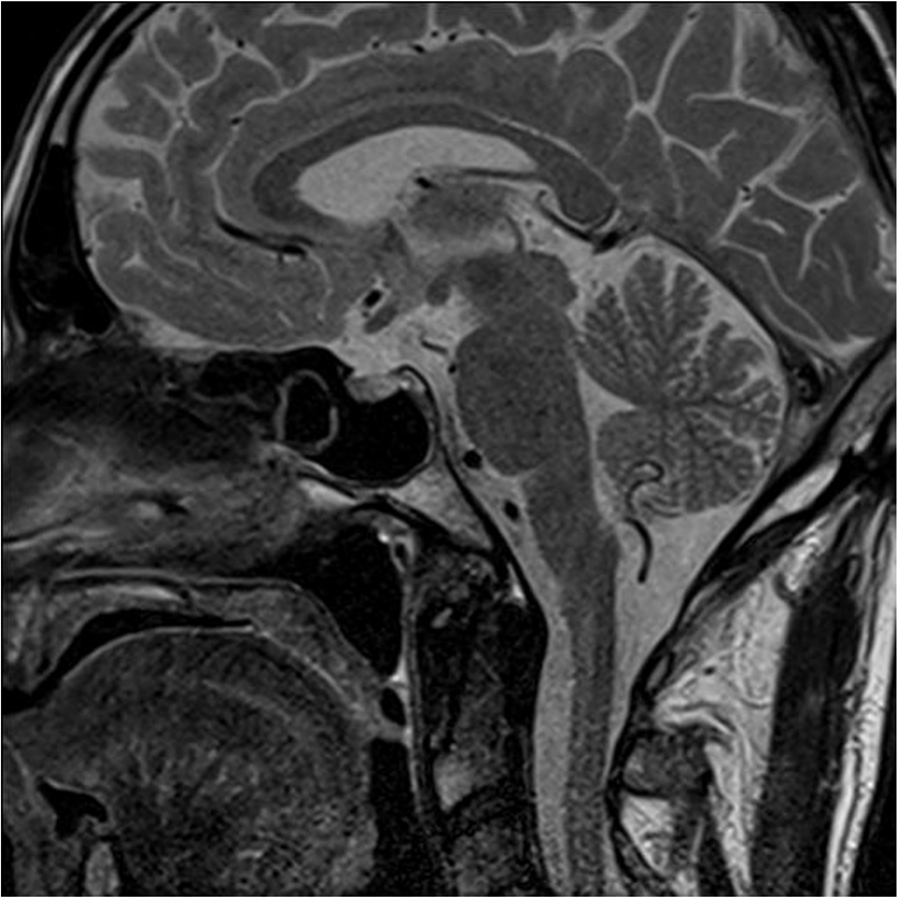
Post-contrast T2-weighted MR-image after several dosis of nivolumab reveals slight diffuse enlargement of the pituitary gland in the absence of micro- or macroadenoma

**Fig. 2:**
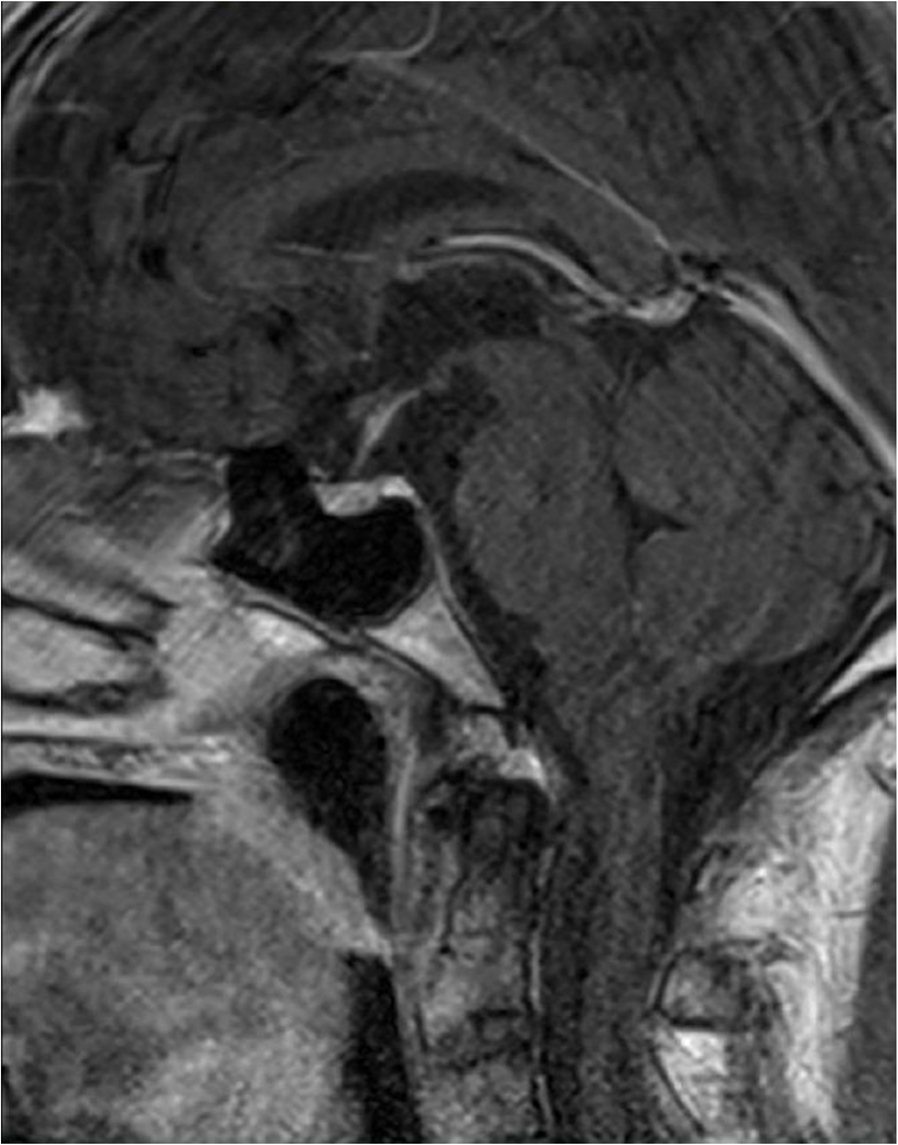
Post-contrast T2-weighted MR-image demonstrates moderately thickened infudibulum, consistent with hypophysitis

Because of suspected adrenal insufficiency we decided to perform stimulation tests, beginning with short ACTH-Test; 250 µg of synthetic ACTH were injected intravenously and blood samples were taken 0, 30 and 60 min after administration. The results were consistent with adrenal insufficiency (Table 2), since cortisol prior to test was low and did not reach the cut-off of 18 µg/dl (< 500 nmol/l). An insulin-induced-hypoglycemia-test followed under strict monitoring-control. The patient fasted for more than 8 h and the test was performed early in the morning. No further hypoglycemia was documented before the test, as already mentioned. Insulin was injected intravenously at the recommended dose of 0.15 IU/kg. Hypoglycemia was documented after 45 Min, with BG levels by 50 mg/dl and the insulin infusion was stopped. As seen in Table 3, ACTH- and Cortisol levels were low (normal range for adults 6–76 pg/ml and 10–20 ug/dl respectively) and despite symptomatic induced-hypoglycemia, a stimulation was not observed. The moderate increase of growth hormone (GH) suggested a mild insufficiency and the diagnosis of a secondary adrenal insufficiency was made. Finally, we performed an MRI imaging, which revealed thickened pituitary stalk, consistent with hypophysitis (Figs. 1 and 2). The patient’s condition gradually improved after administration of hydrocortisone, with normalisation of sodium levels. The patient was discharged from the hospital on 30 mg hydrocortisone daily. He was provided with all necessary information and instructions, as well as emergency hydrocortisone injection kit prior to discharge.

**Table 2:** Short ACTH-Test after administration of 250 ug synthetic ACTH intravenously

**Time (Min.)**	**00**	**30**	**60**
Cortisol (ug/dl)	0.5	2.74	3.19

The Table shows time after administration in minutes (Min.) as well as serum cortisol levels in ug/dl

**Table 3:** Insulin-induced- hypoglycaemia –test

**Time (Min.)**	**-30**	**0**	**15**	**30**	**45**	**60**	**90**	**120**
ACTH (pg/ml)	1	1	< 1	1.2	1.1	1.0	< 1	< 1
Cortisol (ug/dl)	1	1	1	1	0,9	1	1	1
STH (ng/ml)	-	0.5	0.5	0.2	2.2	5.6	6.3	8.4

*ACTH* Adrenocorticotropic hormone, *STH* Somatotropin hormone

## Discussion

We reported a patient with secondary adrenal insufficiency, as a result of a nivolumab-induced hypophysitis that manifested 6 months after treatment discontinuation. Several cases with nivolumab-induced hypophysitis have been reported, all during treatment. One case reported late-onset adrenal insufficiency after treatment termination, without concomitant hypophysitis [10]. Nivolumab is a monoclonal antibody, also known as an immune checkpoint-inhibitor. It improves overall survival as well as progression-free survival, when used in treatment of many tumour types, such as renal-cell carcinoma, melanoma and non-small cell lung cancer [11]. It targets the programmed death-1 (PD-1) receptor and its ligand (PD-L1), which is expressed in many tumour cells as well as T lymphocytes. This interaction leads to functional exhaustion of a cytotoxic immune response, since the blockade of the PD-1 receptor activates T cells to eliminate cancer cells [12].

Although these agents are clinically highly effective, several side effects affecting the endocrine system have been reported, the most common being nivolumab-induced thyroiditis, followed by hypophysitis and rarely adrenalitis [13]. Interestingly, hypophysitis appears to be more common after use of another checkpoint inhibitor, ipilimumab (an antibody against cytotoxic T- lymphocyte–associated antigen 4 [CTLA-4]), alone or in combination with nivolumab. Incidence is estimated between 0.8 and 15 % and seems to be dose-related, while male sex and increasing age seem to be relevant risk factors [14]. On the other hand, immune-related hypophysitis after nivolumab monotherapy is extreme rare (< 1 %). Recent reviews confirm that immune-related hypophysitis is most common with single-agent anti-CTLA-4, followed by the combination with anti- PD-1 [15]. Immune-related hypophysitis with nivolumab monotherapy on the other hand appears to be extreme rare. Since none of the published cases of hypophysitis has been confirmed through biopsy findings, little is known for the exact mechanism. Iwama et al. report that endocrine cells of pituitary, mostly prolactin- and TSH cells also might express CTLA-4 proteins [16]. As a result, the administration of a CTLA-4 blocking antibody, such as ipilimumab, could trigger immune reactions leading to complement activation and subsequent pituitary infiltration via T-cell and antibody dependent mechanisms. On the contrary, nivolumab, as an IgG4-based monoclonal antibody does not activate complement pathway and tissue inflammation as effectively as like CTLA-4 [3] and there is no clear evidence that pituitary expresses PD-1 either [17]. This possibly explains the lower rate of hypophysitis related to these agents. A recent study with 25 patients observed over a longer period, concluded that ACTH deficiency appears usually 28 weeks after their administration and is most likely definitive, while multiple deficiencies appear to be less common. It has been hypothesized that, in PD1/PDL1-induced hypophysitis, the development of antibodies against ACTH or even HLA predisposition might have an additive role. However, MRI scans are performed months after the diagnosis is made, therefore missing the enlargement phase, which occurs after 1 month [18].

Autoimmune hypophysitis is a chronic inflammation of the pituitary gland [2] and is classified by location (adenohypophysitis, infundibulo-neurohypophysitis, or panhypophysitis), histopathological findings (mainly granulomatous or lymphocytic) or etiology. While primary hypophysitis refers to non-identified cause, secondary hypophysitis is mostly due to systemic inflammatory disease or inflammatory response to pharmacological agents, such as cancer immunotherapy [19].

MR Imaging is the best technique to assess patients with suspected hypophysitis, helping to exclude other pathological conditions, such as neoplastic lesions and adenomas. Typical findings suggest hyperintensity on T1-weighted sequence, diffuse symmetric enlargement and thickened pituitary stalk above 4 mm, often with V-shape appearance [20]. Interestingly, the absence of contrast enhancement of the posterior gland might be indicative for the presence of diabetes insipidus [21]. Faje et al. reported that even before the onset of clinical symptoms, pituitary enlargement can be present [22]. Importantly, normal MRI- present in 25 % of cases- does not exclude hypophysitis [23]. Observed neuroradiological abnormalities usually resolve within 2 months [13]. In our case, both mild enlargement of the gland as well as thickened stalk were observed.

Our patient presented with fatigue and anorexia resulting from an isolated ACTH-deficiency following treatment with nivolumab. To our knowledge there is only one case reporting isolated secondary adrenal insufficiency without hypophysitis after treatment termination, since most of the cases occurred during treatment. The authors conclude that nivolumab effects last for long time and side effects might indeed appear even after drug discontinuation [10]. Since no other cause could be identified, we assume that nivolumab can trigger a late response due to progressive destruction before symptoms become overt. Hypothyroidism and secondary adrenal deficiency, occurring also separately, are the most common endocrinopathies observed, while gonadal dysfunction and diabetes insipidus are rare, again mostly after ipilimumab [8]. A few cases from literature report that patients suffering from hypophysitis often remain asymptomatic, while for others report symptoms ranging from mild headaches and fatigue to even acute apoplexy [24]. Recently, Inglesias et al. reported that isolated ACTH deficiency occurs more frequently after nivolumab treatment for melanoma, affecting mostly men. Fatigue and anorexia are the most common symptoms, while laboratory measurements reveal hyponatriemia, as in our case- and eosinophilia. Nevertheless, MRIs might be normal [25]. Detailed medical history and clinical investigation, can aid diagnosis, even before typical signs and symptoms occur. Notably, some authors suggest laboratory investigation prior to each treatment cycle during the first 16 weeks of treatment, in order to detect both early and late-onset cases.

Our patient’s condition improved rapidly after glucocorticoid administration. Symptomatic treatment with glucocorticoid is the current suggestion, which, unlike thyroid or gonadal deficiencies, might be permanent [26]. Immunosuppressive agents, such as azathioprine, have been suggested by some authors, but there is only limited experience. Pituitary surgery is rarely necessary. [27]. Hormone replacement treatment should be started if needed, after careful blood analysis. GH-deficiency, like in our patient, should not be treated because of underlying malignancy. Current guidelines recommend that cancer immunotherapy for mild cases of hypophysitis should not be necessarily withdrawn. The decision should be made after careful consideration of the impact on the progression-free survival of the underlying malignancy.

We conclude that even after discontinuation of nivolumab treatment, hypophysitis with isolated ACTH-deficiency might occur. Due to increasing use of immune-checkpoint inhibitors, physicians as well as patients should be aware of potential side effects involving endocrine organs. Constant clinical assessment and routine control are needed to prevent adrenal crisis. Since literature suggests partial recovery of pituitary function, endocrine follow-up and lifelong steroid treatment might be necessary.

## Data Availability

Not applicable.

## Abbreviations

ACTH: Adrenocorticotropic hormone
BG: Blood glucose
CTL-4: Cytotoxic T-lymphocyte antigen-4
DHEAS: Dehydroepiandrosterone sulfate
FSH: Follicle-stimulating hormone
FT4: Free thyroxine
GFR: Glomerular filtration rate
GH: Growth hormone
IGF-1: Insulin-like growth factor 1
LH: Luteinizing hormone
MRI: Magnetic resonance imaging
Osm: Osmolarity
PD-1: Programmed death-1
SHBG: Sex hormone binding globulin
STH: Somatotropin hormone
TgAb: Antithyroglobulin antibodies
TPOAb: Anti-thyroid peroxidase antibodies
TSH: Thyroid stimulating hormone
